# miR-30a/SOX4 Double Negative Feedback Loop is modulated by Disulfiram and regulates EMT and Stem Cell-like properties in Breast Cancer

**DOI:** 10.7150/jca.57752

**Published:** 2021-06-16

**Authors:** Zijian Liu, Mi Mi, Xin Zheng, Caijiao Zhang, Fang Zhu, Tao Liu, Gang Wu, Liling Zhang

**Affiliations:** Cancer Center, Union Hospital, Tongji Medical College, Huazhong University of Science and Technology, Wuhan, China.

**Keywords:** breast cancer, epithelial-mesenchymal transition, cancer stem cell, miR-30a, SOX4

## Abstract

**Background:** Both epithelial-to-mesenchymal transition (EMT) and cancer stem cells play important roles in development and progression of breast cancer. MicroRNA (miR)-30 family members have been reported to be associated with the regulation of EMT and stem cell phenotypes, however, the underlying molecular mechanisms are not well understood.

**Methods:** miR-30a stable transfectants of breast cancer cell lines were created using a lentiviral system. Bioinformatics analysis was performed to explore miR-30a target genes and SOX4 was selected and identified by dual luciferase reporter assay. The effects of miR-30a and target gene SOX4 on EMT and CSC phenotypes in breast cancer were explored *in vitro* and *in vivo*.

**Results:** Overexpression of miR-30a in breast cancer cells inhibited EMT and CSC phenotypes by targeting SOX4. Luciferase reporter assay confirmed that miR-30a directly targeted 3'UTR of SOX4, and formed a double-negative feedback loop with SOX4. Functional experiments demonstrated that knockdown of SOX4 suppressed EMT and CSC phenotypes of breast cancer cells through TGF-β/SMAD pathway, which was consistent with the inhibitory effects by overexpression of miR-30a. Additionally, we found disulfiram can upregulate miR-30a expression, and high miR-30a expression was associated with a good prognosis in breast cancer patients through TCGA database.

**Conclusion:** Our findings suggest a novel double-negative loop between miR-30a and SOX4 mediated regulation of EMT and CSC features in breast cancer through TGF-β/SMAD pathway, highlighting a novel therapeutic target for breast cancer.

## Introduction

Breast cancer is a malignant tumor with the highest morbidity and mortality among women worldwide, which alone accounts for 30% of female cancers, and approximately 48,530 cases of ductal carcinoma *in situ* of the female breast will be newly diagnosed in 2020 1. Although advances in systemic and comprehensive therapy, especially molecular targeted therapy, have significantly improved the survival of breast cancer patients, the median survival time of patients with metastatic breast cancer is still only 2-3 years 2. Therefore, it is of great influence to further investigate the mechanisms of tumorigenesis and development, and search for new therapeutic molecules, which will possess important theoretical and practical significance of formulating new therapeutic strategies.

Epithelial-mesenchymal transition (EMT) is thought to play an important part in the development and metastasis of epithelial-derived malignancies, and caused poor histologic differentiation, local invasiveness, distant metastasis and drug resistance in various cancer especially breast cancer 3. Loss of epithelial marker E-cadherin expression and gain of mesenchymal marker vimentin are considered to be the most important molecular characteristics of EMT 4, and upregulation of EMT-related transcription factors (EMT-TF), such as Snail1 and ZEB1 could also foster the maintain of EMT process, however, the downregulation or deletion of EMT-TF might reverse it 5. Cancer stem cell is characterized by its high tumorigenicity, multidrug-resistant protein expression, differentiation potential, and, most importantly, its ability to self-renew, which could also play a very important role in the development and progression of breast cancer 6. More and more evidence 7 and our previous study 8 have shown that EMT is closely associated with tumor stem cells, and the process of EMT can produce cells with stem cell-like (CSC) characteristics and self-renewal ability. EMT and CSC are interwoven and closely related, which is a reasonable entry point to explore the mechanism of tumor progression and treatment strategies.

MicroRNAs (miRNAs) are a class of noncoding RNA with a length of 19-22 nucleotides, which could suppress gene expression by forming base pairs with target mRNAs and thereby regulate various tumor related biological progression implicated in cancer occurrence and development 9, 10. There are many miRNAs involved in the regulation of breast cancer EMT and CSC have been reported including miR-200, miR-205, miR-155, let-7 and miR-30 11, 12, among them miR-30a high expression has been shown to be a good prognostic factor for breast cancer. Up-regulated expression of miR-30a in tumor cells can target and inhibit EMT-related molecules (such as Snail, Slug, ZEB2, Vimentin, etc.) 13, 14, as well as Ubc9 and self-renewal ability of tumor stem cells 15. Although miR-30a has shown negative regulatory effects on EMT and CSC, its mechanism has not been fully clarified and needed further exploration.

In this study, several common miRNA target prediction tools (DIANA, miRTarBase, TargetScan, miRanda and mirDB) were used to identify novel miR-30a target genes, and then the intersection genes predicted by the above database were delivered to Gene Ontology (GO) enrichment analysis. Combined with bioinformatics analysis via TCGA database and basic experiments, transcription factor SRY-box transcription factor 4 (SOX4), which has been reported to be upregulated in breast cancer 16 and another recently discovered key molecule in the TGF-β pathway directly targeted by TGF-β 17, was selected for further investigation. With a series of experiments, we eventually elucidated that miR-30a targeted the 3'UTR of SOX4 and formed a double-negative loop with SOX4 to inhibit EMT and stem cell-like phenotypes in breast cancer via blockade of TGF-β/SMAD signaling pathway both *in vitro* and *in vivo*. Additionally, we found disulfiram, an anti-alcoholism drug, can upregulate miR-30a expression, and high miR-30a expression was identified as a good prognostic factor for breast cancer patients through TCGA database. Our results might improve the interpretation of molecular mechanism of inhibition effects of miR-30a on EMT and stem cell-like properties, and thereby provide a novel therapeutic target for breast cancer treatment.

## Methods

### Cell lines and culture

Human breast cancer cell lines MCF-7, MDA-MB-231, BT-549, SK-BR-3, MDA-MB-468 and T-47D and normal breast cell line HBL-100 were purchased from Cell Bank of Type Culture Collection of the Chinese Academy of Sciences (Shanghai Institute of Cell Biology, Shanghai, China). The cell lines MCF-7, MDA-MB-231, SK-BR-3, T-47D and HBL-100 were cultured in Dulbecco's modified Eagle medium (DMEM, Gibco, Thermo Fisher Scientific, MA, USA) supplemented with 10% heat-inactivated fetal bovine serum (FBS, Gibco, Thermo Fisher Scientific, MA, USA). BT-549 and MDA-MB-468 were cultured in Roswell Park Memorial Institute 1640 medium (RPMI164, Gibco, Thermo Fisher Scientific, MA, USA) supplemented with 10% FBS. All cell lines were cultured in a humidified incubator containing 5% CO_2_ at 37 °C.

### Reagents and antibodies

The details for primary antibodies was listed in Supplementary Table 1. Disulfiram (DSF) were purchased from Sigma-Aldrich (Sigma, Merck, Darmstadt, Germany) and dissolved in Dimethyl sulfoxide (DMSO). TGF-β (Peprotech, Rocky Hill, NJ, USA) was dissolved in 10 mM citric acid, concentration of 10 ng/mL to induce cells.

### Western blot

After RIPA protein extraction reagent supplied with PMSF and phosphorylated protease inhibitor treatment, extracted proteins were separated on a 10% SDS-PAGE and then transferred to polyvinylidene fluoride (PVDF) membrane. The membranes were blocked with 5% nonfat milk in 0.1% TBST for 1 h in room temperature and then incubated with the specific primary antibodies over night at 4 °C, followed by incubation with secondary antibodies (1:4000 dilution, Boster, Wuhan, China). The protein bands were visualized by SuperSignal West Pico Chemiluminescence Substrate (Peptbio, Wuhan, Hubei, China).

### Quantitative reverse transcription-PCR

Total RNA was extracted using Trizol reagent (Invitrogen, Carlsbad, CA, USA). Following quantification by NanoDrop 2000 (Thermo Fisher Scientific, Waltham, MA), the extracted total RNA was reverse transcribed using Reverse Transcription Kit (TaKaRa, Dalian, Liaoning, China). Quantitative reverse transcription-PCR (qRT-PCR) assays were performed using SYBR Premix Ex Taq Kit (TaKaRa, Dalian, Liaoning, China) on StepOne Real-time PCR system (Applied Biosystems, Foster City, CA, USA). U6 was used as an internal control for miR-30a and GAPDH served for SOX4. The specific primer sequences (Supplementary Table 2) synthesized by Shanghai Sangon Biological Engineering Technology and Service. The relative expression level was calculated using the 2^-ΔΔCt^ method. The experiments were performed in biological triplicates.

### Immunofluorescence staining

Cells were fixed with 4% paraformaldehyde solution for 30 min and permeabilized with 0.1% Triton X-100/PBS. After blocked with 5% BSA for 30 min, cells were incubated with primary antibodies overnight at 4 °C. After washed with PBS, followed by incubation with Cy3-conjugated goat anti-rabbit or anti-mouse IgG secondary antibodies (1:200 dilution, Boster, Wuhan, China) in the dark for 1 h at 37 °C, counterstained with DAPI. Images were observed under an Olympus Fluoview FV1000 laser-scanning confocal microscope.

### Mammosphere formation assay

The cells were collected and furthered cultured in ultra-low attachment plates (Corning, MA, USA) in 2mL serum-free DMEM/F12 supplemented with B27 (Invitrogen, Carlsbad, CA, USA), 20 ng/mL basic fibroblasts growth factor (bFGF, Peprotech, Rocky Hill, NJ, USA), 20 ng/mL epidermal growth factor (EGF, Peprotech, Rocky Hill, NJ, USA), 4 mg/mL insulin (Sigma, Merck, Darmstadt, Germany) at a density of 1000 cells/well. Fresh medium was supplemented every 3 days. After 10-14 days culture, the mammospheres were counted and photographed under inverted microscope.

### Flow cytometry assay

The cells (1 × 10^6^/mL) were suspended in PBS containing 2% FBS (FACS buffer), and then incubated with CD44-PE and CD24-FITC (BD Biosciences, Franklin Lakes, NJ, USA) on ice in 4 °C for 30 min kept in the dark place. ALDEFLUOR kit (StemCell Tech., Shanghai, China) was used to detect the ALDH-positive population in cells. The cells (1 × 10^6^/mL) were staining in ALDH substrate containing assay buffer for 30 min at 37 °C. The negative control was treated with a specific ALDH inhibitor diethylaminobenzaldehyde (DEAB). Cells were washed and resuspended in 500 μL FACS buffer and analyzed using a FACS Calibur Flow Cytometer (BD Biosciences, Franklin Lakes, NJ, USA).

### Viral production and cells transfection

Lentiviruses were obtained from Genecopoeia (Rockville, MD, USA) and Obio Technology (Shanghai, China). The cells were seeded into 6-well plates, followed by addition of 1 × 10^8^ TU/mL lentivirus. Stable cell lines for overexpression of miR-30a and knockdown of SOX4 were selected with 2 μg/mL puromycin, for miR-30a inhibitor and overexpression of SOX4 were selected with 200 μg/mL hygromycin and 10 μg/mL blasticidin, respectively.

### Dual luciferase reporter assay

The potential binding sites or mutant 3'-untranslated regions (UTR) fragment of SOX4 were cloned into the GV272 vector (GenChem, Shanghai, China). Luciferase reporters were co-transfected with pre-miR-30a mimics or negative control and wild-type or mutant 3'-UTR-luc by jetPRIME (Polyplus‐transfection, France). At 48 h after transfection in HEK293T cell, the luciferase activities were detected with the Dual‐Luciferase Reporter Assay System (Promega, Madison, WI, USA) in Synergy H1 system. TRAF6 and miR-146b 18 were selected as positive control to test whether the transfection system was successfully constructed. Each experiment was performed in triplicates.

### Tissue specimens and immunohistochemistry

A total of 42 breast cancer specimens were obtained retrospectively from the Department of Pathology, Union Hospital. The study was approved by the local Ethics Committee, and informed consent was obtained from each patient. Tissue microarray (TMA) construction, including 133 breast cancer specimens, were created by contract service at Shanghai OUTDO Biotech, China. And the clinical information for TMA was shown in Supplementary Table 3. Immunohistochemistry (IHC) staining was performed in formalin-fixed paraffin-embedded tumor tissue sections. The sections were stained with standard procedures and examined under a fluorescence microscope. The immunoreactivity score (IRS) of SOX4 was evaluated to four levels (negative, weak, moderate and strong staining) by the intensity of immunostaining and the percentage of immunoreactive cells.

### Tumor xenograft experiments

Five-week-old female BALB/c nude mice (Beijing HFK Bioscience, Beijing, China) were housed under pathogen-free conditions according to the animal care guidelines of Huazhong University of Science and Technology. The animal experiments were reviewed and approved by the local Ethical Committee. The mice were randomly subdivided into two groups (8 mice/group), and each group was injected subcutaneously in the right flank of mice a total of 1 × 10^7^ stable transfection MCF-7 cells and their negative control cells, respectively. Giving that the characteristics of MCF-7 cells and preliminary experimental results, MCF-7 tumor growth was sustained by estrone administered at 1 mg/l in the animals' water supply. Tumor dimensions was measured every three days using a Vernier caliper and tumor volumes were calculated according to the formula: length × width^2^ × 0.5. Forty days later, the mice were sacrificed, and tumors were collected and photographed.

### Bioinformatics analysis

The gene expression profiles of 973 breast cancer samples were downloaded from TCGA database. Gene Set Variation Analysis (GSVA) was resorted to reveal the pathways enrichment among samples via GSVA_1.30.0 package in R 19 to evaluate t score and assign pathway activity conditions. Five databases were used including DIANA 20, miRTarBase 21, TargetScan 22, miRanda 23, and mirDB 24 to predict candidate mRNAs of interest that can be targeted by miRNAs.

### Statistical analysis

For experimental data, continuous values were described with mean ± standard error (SE) and tested by one way ANOVA. The χ^2^ test was applied to determine the significance of SOX4 staining in breast cancer patients with different subtypes. Kaplan-Meier survival analysis was performed and tested by Log-rank test. Wilcoxon test was applied to determine the significance of different groups' expression of SOX4 and miR-30a in TCGA dataset. Error bars represent SE for all figures. Statistical significance was defined as follows: *, *P* <0.05; **, *P* <0.01; ***, *P*<0.001.

## Results

### miR-30a overexpression inhibits EMT and stem cell-like phenotypes

To verify the differential expression of miR-30a in breast cancer cells, qRT-PCR was performed in a panel of cell lines, including MDA-MB-231, MCF-7, MDA-MB-468, BT-549, SK-BR-3, T-47D, and normal breast cell HBL-100. The results showed that miR-30a expression was significantly lower in breast cancer cell lines than normal breast cells (Figure S1A), especially in MDA-MB-231 and MCF-7 cell lines. To investigate the effects of miR-30a on EMT and stem cell-like features, we overexpressed miR-30a in MDA-MB-231 and MCF-7 cells (Figure S1B).

**Figure 1:**
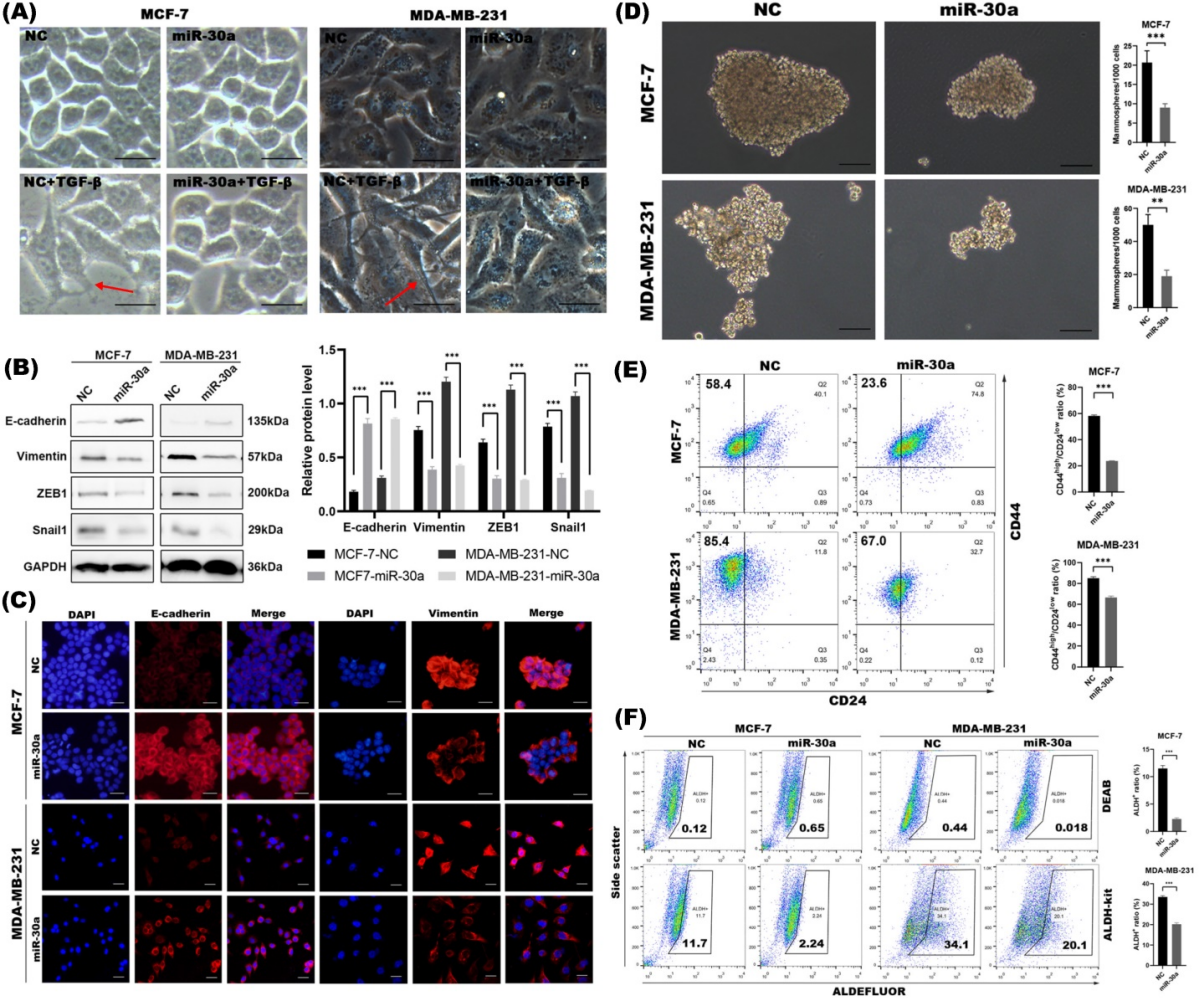
Overexpression of miR-30a inhibited EMT and CSC phenotypes in breast cancer cells. **(A)** Morphological changes of cells with or without miR-30a overexpression and photographed under inverted microscope. EMT morphological changes were induced by TGF-β. Red arrows point to typical morphological changes by TGF-β. Scale bar, 100 µm. **(B)** Western blot of expression for epithelial marker E-cadherin, mesenchymal markers vimentin and EMT-TFs Snail1 and ZEB1. **(C)** Immunofluorescence staining for the epithelial and mesenchymal markers. Scale bar, 100 µm. **(D)** Phase contrast images of mammospheres formation. Scale bar, 100 µm. **(E)** Flow cytometry assay of breast cancer stem cell markers CD44 and CD24. **(F)** ALDEFLUOR assay. The error bars indicate the standard errors of the mean from three independent experiments. ** P < 0.01, *** P < 0.001.

In order to elucidate how cell morphology changes during EMT process with or without miR-30a overexpression, cells were treated with TGF-β, a classical EMT inducer. We found that breast cancer cells lost adhering “cobblestone” shape and elongated with pseudopods by TGF-β, while overexpression of miR-30a could partly prevent EMT morphological change induced by TGF-β and retained their cobblestone-like epithelial morphology with tight cell-cell adhesion (Figure 1A). Western blot (Figure 1B) and immunofluorescence staining (Figure 1C) showed that ectopic expression of miR-30a potently upregulated E-Cadherin and downregulated vimentin. miR-30a overexpression also inhibited the expression of EMT transcriptional factors (EMT-TFs) ZEB1 and Snail1. In order to make our experiment more rigorous, we selected HBL-100, which intrinsically highly expressed miR-30a, to transfect miR-30a inhibitor lentivirus (miR-30a inhibitor), which was designed to strongly inhibit the endogenous mature miR-30a expression (Figure S1C). The results revealed that the functional inhibition of miR-30a could lead to loss of E-cadherin and upregulated of vimentin, Snail1 and ZEB1 (Figure S1D). These data suggested that miR-30a overexpression potently reversed EMT phenotype.

Increasing evidence has proven that EMT program is closely linked with stemness and generates cells with stem cell-like (CSC) properties 25. Since we found miR-30a potently inhibited EMT phenotype, we further investigated whether miR-30a also inhibit CSC. Mammosphere formation assay indicated that the overexpression of miR-30a reduced mammospheres both in size and in number when compared with the negative control cells (Figure 1D). Meanwhile, increased expression of miR-30a reduced the sorted CD44^high^/CD24^low^ cells subpopulation (Figure 1E) and ALDH^+^ cells population (Figure 1F). Taken together, miR-30a overexpression could inhibit EMT and CSC phenotypes in breast cancer cells.

In order to better verify previous studies and carry out our follow-up research about the biological function of miR-30a underlying in the breast cancer, we have downloaded the expression profile data from TCGA database containing a total of 973 breast cancer patients and 104 normal samples. After our analysis on these public data, we found that the basic expression of miR-30a was significantly downregulated in breast cancer groups compared with normal breast tissues (Figure S1E).

### SOX4 is a direct target of miR-30a in breast cancer cells

**Figure 2:**
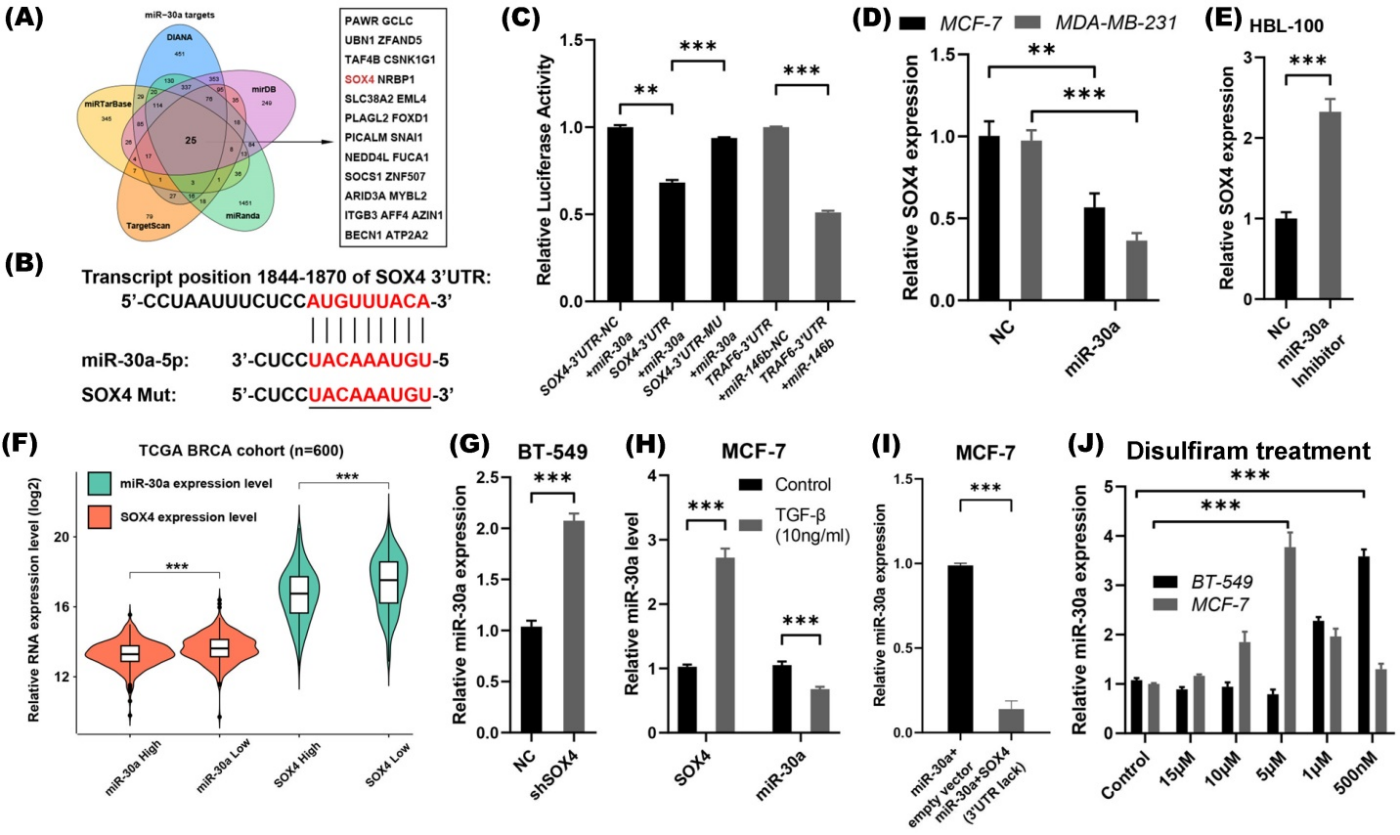
SOX4 is a direct target of miR-30a and a double-feedback loop formed between SOX4 and miR-30a. **(A)** Prediction of miR-30a target genes and the 25 intersection genes were shown.** (B)** The binding site of miR-30a-5p in the 3′-UTR region of SOX4 mRNA and the mutant SOX4 3'-UTR sequence for luciferase assay.** (C)** Luciferase reporter assay. Wild type and mutant type of SOX4 3'-UTR luciferase constructs containing miR-30a binding site were cloned and then co-transfected with miR-30a precursors. **(D)** Relative expression of SOX4 was detected by qRT-PCR in MCF-7 and MDA-MB-231 cells that were stably expressed miR-30a. **(E)** Relative expression of SOX4 was detected in HBL-100 cells transfected with miR-30a inhibitor. **(F)** The expression of miR-30a and SOX4 were calculated in different groups. **(G)** Relative expression of miR-30a in shSOX4 BT-549 cells compared with control group. **(H)** Relative expression of miR-30a and SOX4 in MCF-7 cells treated with 10 mg/mL TGF-β after 24 h. **(I)** Relative expression of miR-30a in cells transfected with miR-30a or co- transfected with miR-30a and SOX4 lacking 3'UTR. *** P < 0.001. **(J)** The relative expression of miR-30a were tested in MCF-7 and BT-549 cell lines treated with different disulfiram concentrations. The error bars indicate the standard errors of the mean from three independent experiments. ** P < 0.01, *** P < 0.001.

In order to investigate the mechanism through which miR-30a negatively regulated the EMT and CSC phenotypes, we searched for targets of miR-30a using several target prediction algorithms databases, including DIANA, miRTarBase, TargetScan, miRanda and mirDB. After taking the intersection of those predicted target molecules, only 25 genes were suggested common target genes of miR-30a (Figure 2A). Among of these genes, SOX4 has been reported as a crucial regulator of EMT 26, therefore, we chose SOX4 as an interested gene to further explore.

Consistent with findings of previous studies 27, 28, the luciferase reporter assay also confirmed that SOX4 is a direct target of miR-30a (Figure 2B-C). In miR-30a stable expression MCF-7 and MDA-MB-231 cells, SOX4 mRNA was significantly decreased (Figure 2D). Meanwhile, in HBL-100 cells transfected with miR-30a inhibitor lentivirus, SOX4 mRNA was significantly increased (Figure 2E). Collectively, miR-30a directly targeted SOX4, and repressed its expression.

### miR-30a and SOX4 form a double negative-feedback loop modulated by disulfiram

The relationship between SOX4 and miR-30a was analyzed through breast cancer TCGA database (n=973). The expression of genes was sorted by values. The first 300 samples were defined as high expression, and the last 300 samples were defined as low expression. The results showed that SOX4 expression was lower in miR-30a high expression group than those in miR-30a low expression group, and vice versa (Figure 2F). Considering the reciprocal expression patterns of miR-30a and SOX4 in breast cancer samples, feedback circuit could exist between miR-30a and SOX4.

To investigate whether SOX4 inhibit the expression of miR-30a, expression of SOX4 in breast cancer cell lines were screened, and BT-549 cell line presented a high level of endogenous SOX4 expression (Figure S2). Knockdown of endogenous SOX4 in BT-549 by shRNA induced expression of miR-30a (Figure 2G). On the other hand, overexpression of SOX4 induced by TGF-β (10 ng/mL) suppressed the expression of miR-30a (Figure 2H), suggesting that miR-30a might be a target of SOX4. What's more, we directly overexpressed the full-length SOX4 cDNA (lacking the 3'-UTR) in the stably expressing miR-30a MCF-7 cells (Figure S3A). Meanwhile, we found that miR-30a was significantly down-regulated in the combinational transfection panels, indicating that overexpressed SOX4 could suppress the expression of miR-30a (Figure 2I). Taken together, miR-30a and SOX4 could inhibit each other and formed a double negative feedback loop.

As our previous study reported, disulfiram, an anti-alcoholism drug, could reverse EMT phenotype and suppress stem cell-like properties in breast cancer via inhibition ERK/NFκB/Snail pathway 8. We speculated whether the regulation of miR-30a/SOX4 was involved in the effects of disulfiram. MCF-7 and BT-549 cells were treated by different concentrations of disulfiram, and the results showed that disulfiram increased miR-30a expression at appropriate concentrations (Figure 2J).

### Suppression of SOX4 is required for miR-30a mediated inhibition of EMT and CSC phenotypes in breast cancer

**Figure 3:**
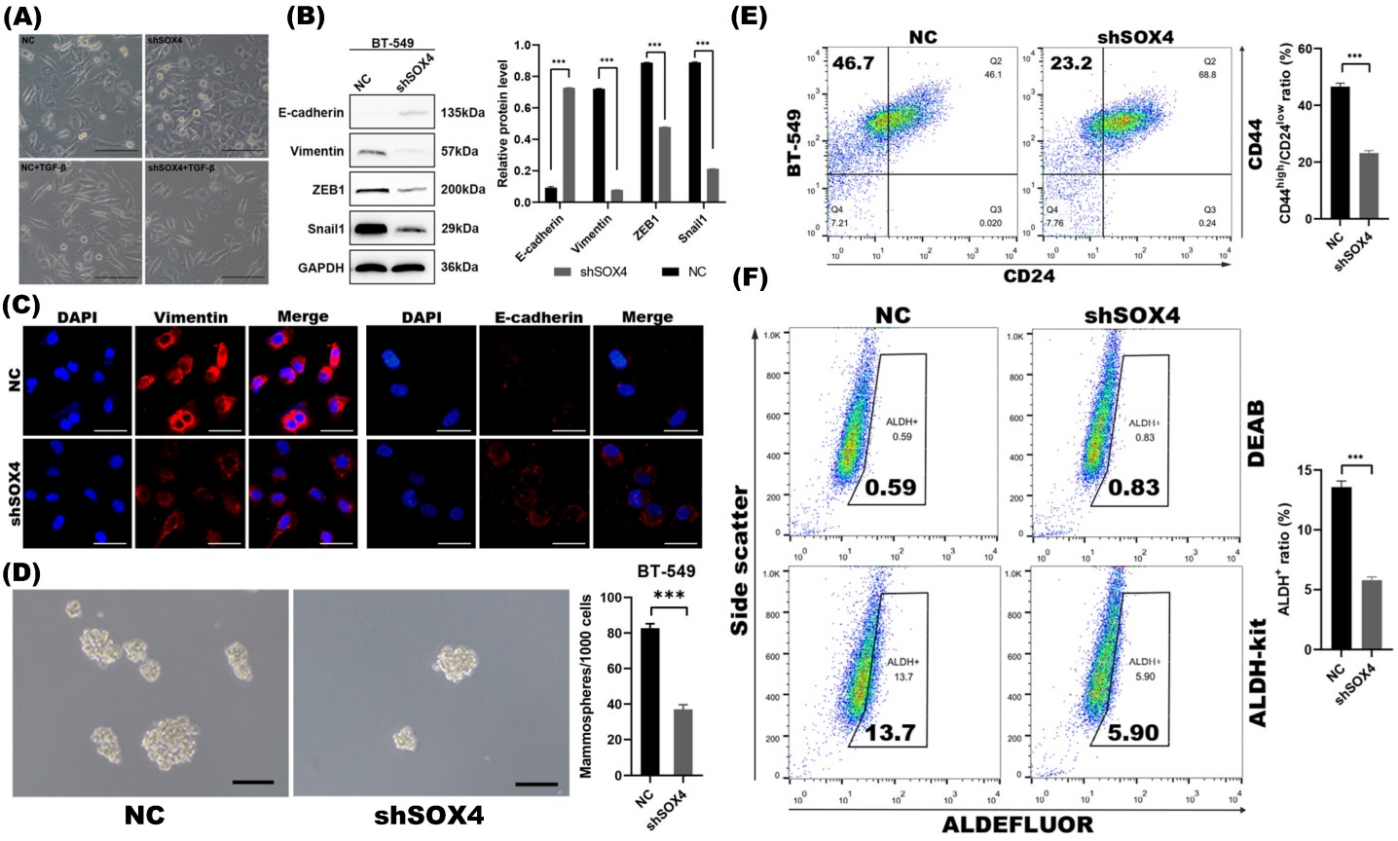
SOX4 knockdown inhibited EMT and CSC phenotype in BT-549 cells. **(A)** Morphological changes of cells with or without SOX4 knockdown. EMT morphological changes were induced by TGF-β. Scale bar, 100 µm. **(B)** Western blot results of EMT related markers. **(C)** Immunofluorescence staining results of EMT related markers. **(D)** Phase contrast images of mammospheres formation. Scale bar, 100 µm. **(E)** Flow cytometry assays of expression of CD44 and CD24 in cells transfected with shSOX4 or empty vector. **(F)** ALDEFLUOR assay. Inhibition of SOX4 significantly decreased ALDH^+^ cell population. The error bars indicate the standard errors of the mean from three independent experiments. *** P < 0.001.

To examine whether the repression of SOX4 by miR-30a mediated EMT and CSC inhibition, we studied the EMT and CSC phenotypes of breast cancer cells following SOX4 knockdown with shRNA. With confirmed knockdown of SOX4 by western blot (Figure S3B) and qRT-PCR (Figure S3C), cells retained their cobblestone-like epithelial morphology during induction of TGF-β (Figure 3A). Results of western blot and immunofluorescence staining revealed that the knockdown of SOX4 reversed EMT program as miR-30a did (Figure 3B-C). The knockdown of SOX4 also suppressed mammospheres formation ability and decreased the proportion of ALDH^+^ and CD44^high^/CD24^low^ subpopulations (Figure 3D-F).

**Figure 4:**
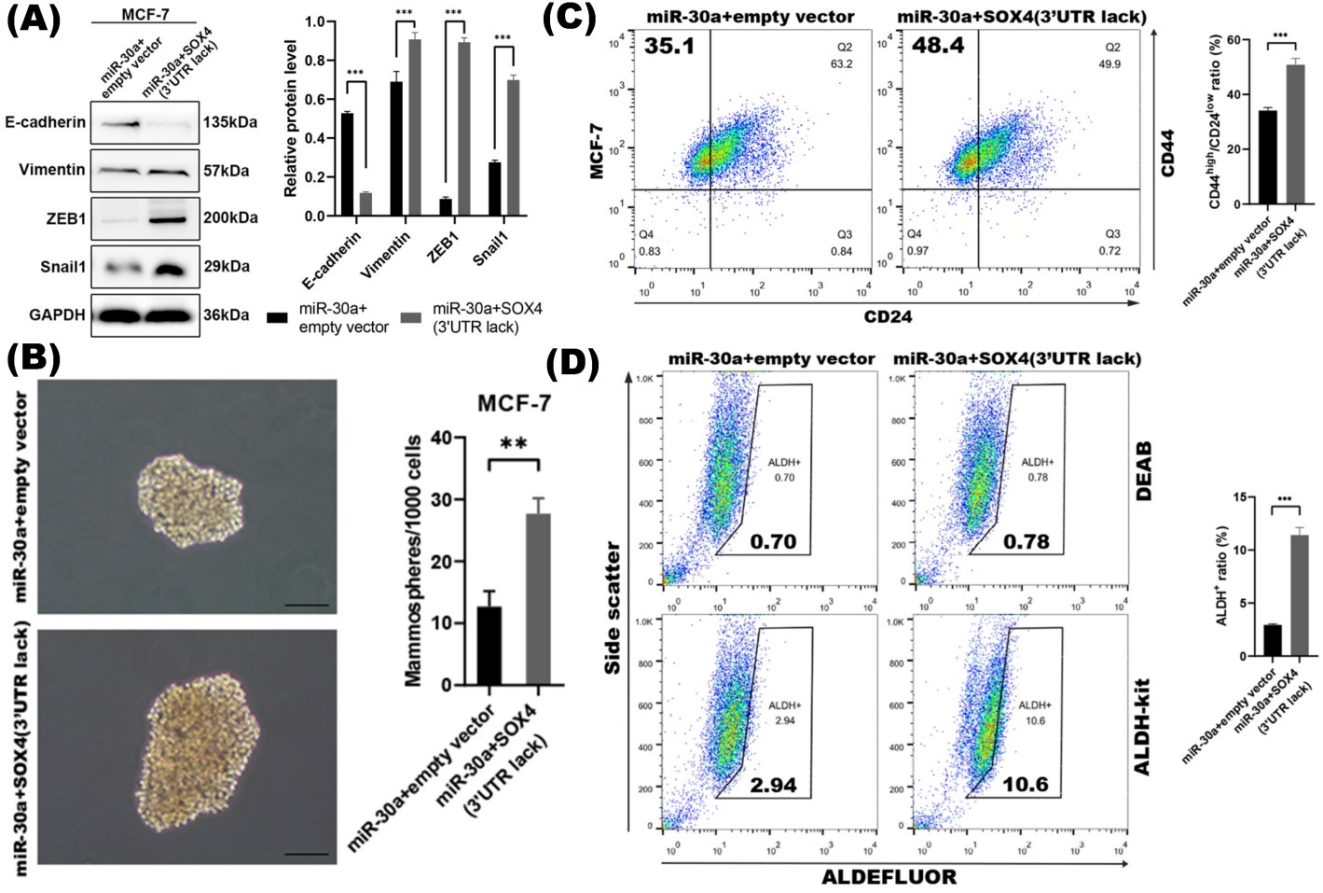
SOX4 was required for miR-30a mediated inhibition of EMT and CSC phenotypes.** (A)** Western blot results of EMT related markers in MCF-7 transfected with miR-30a and SOX4 lacking of 3'UTR.** (B)** Phase contrast images of mammospheres formation. Scale bar, 100 µm. **(C)** Flow cytometry assay of breast cancer stem cell markers CD44 and CD24. **(D)** ALDEFLUOR assay. The error bars indicate the standard errors of the mean from three independent experiments. ** P < 0.01.

To further investigate whether the repression of SOX4 was required for miR-30a mediated EMT and CSC inhibition, we found that the inhibition of EMT and CSC phenotype mediated by the overexpression of miR-30a was abolished by the reintroduction of SOX4. The EMT markers tested by western blot (Figure 4A) and the CSC phenotypes including mammospheres formation ability and proportion of ALDH^+^ and CD44^high^/CD24^low^ subpopulations (Figure 4B-D) were reversed in miR-30a and SOX4 co-expression cells compared with those cells with miR-30a alone. Altogether, these results indicated that suppression of SOX4 was required for miR-30a mediated inhibition of EMT and CSC phenotypes in breast cancer cells.

### miR-30a inhibits EMT and CSC phenotypes through suppression of TGF-β/SMAD pathway in breast cancer

**Figure 5:**
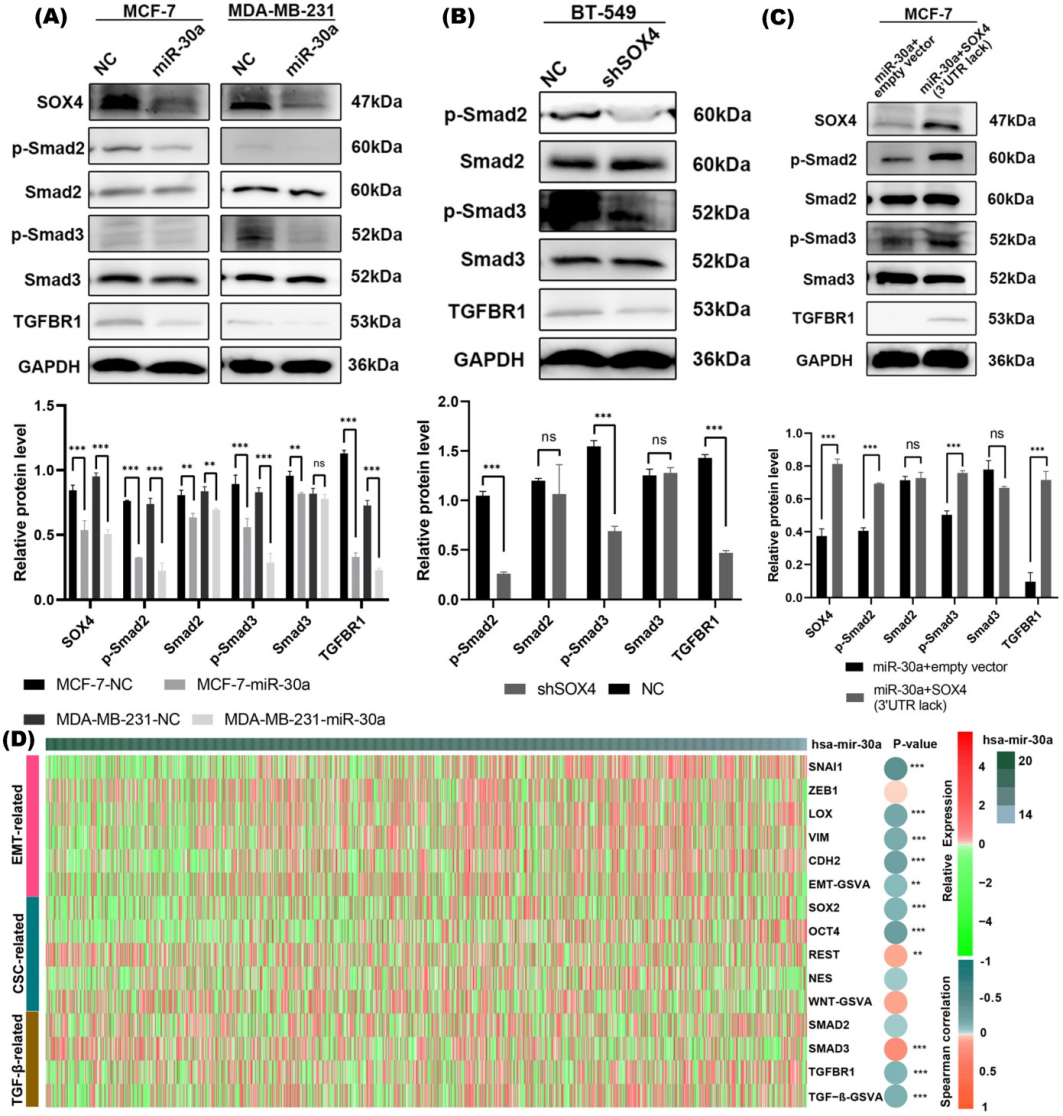
miR-30a inhibited EMT and CSC phenotypes by targeting SOX4 through TGF- in breast cancer cells. **(A)** Western blot results of SOX4 and TGF-β/SMAD pathway related markers in cells with overexpression of miR-30a. **(B)** Western blot results of TGF-β/SMAD pathway related markers in cells with shSOX4. **(C)** Western blot results of TGF-β/SMAD pathway related markers in cells transfected with miR-30a or co-transfected with SOX4 lacking 3'UTR. **(D)** Heat map showed the relationship between miR-30a and markers of EMT, CSC, and canonical TGF-β/SMAD pathway. The color of the heat map represents the level of expression and the depth of the circle indicates the level of Spearman correlation coefficients between the expression of markers and miR-30a. ** P < 0.01, *** P < 0.001.

SOX4 has been reported to be involved in and required for TGF-β induced EMT 16. Our western blot results revealed that overexpression of miR-30a significantly suppressed phosphorylated SMAD2/3 protein (a downstream effector of TGF-β pathway) and TGFBR1 (a receptor of TGF-β) (Figure 5A), while suppression of miR-30a resulted in the upregulation of phosphorylated SMAD2/3 protein and TGFBR1 (Figure S3D). Western blot results revealed that knockdown of SOX4 also inhibited the activation of TGF-β/SMAD pathway (Figure 5B). The suppression of TGF-β/SMAD pathway mediated by overexpression of miR-30a could be reversed by the overexpression of SOX4 (Figure 5C).

Through TCGA database, we further analyzed the relationship between miR-30a and markers of EMT, CSC, and canonical TGF-β/SMAD pathway. The heat map showed an inverse relationship between miR-30a and those related genes (Figure 5D). Taken together, the above results suggested that EMT/CSC- inhibitory effects of miR-30a are mediated, at least in partly, via suppressing TGF-β/SMAD pathway.

### miR-30a inhibits EMT and CSC phenotypes in breast cancer *in vivo*

**Figure 6:**
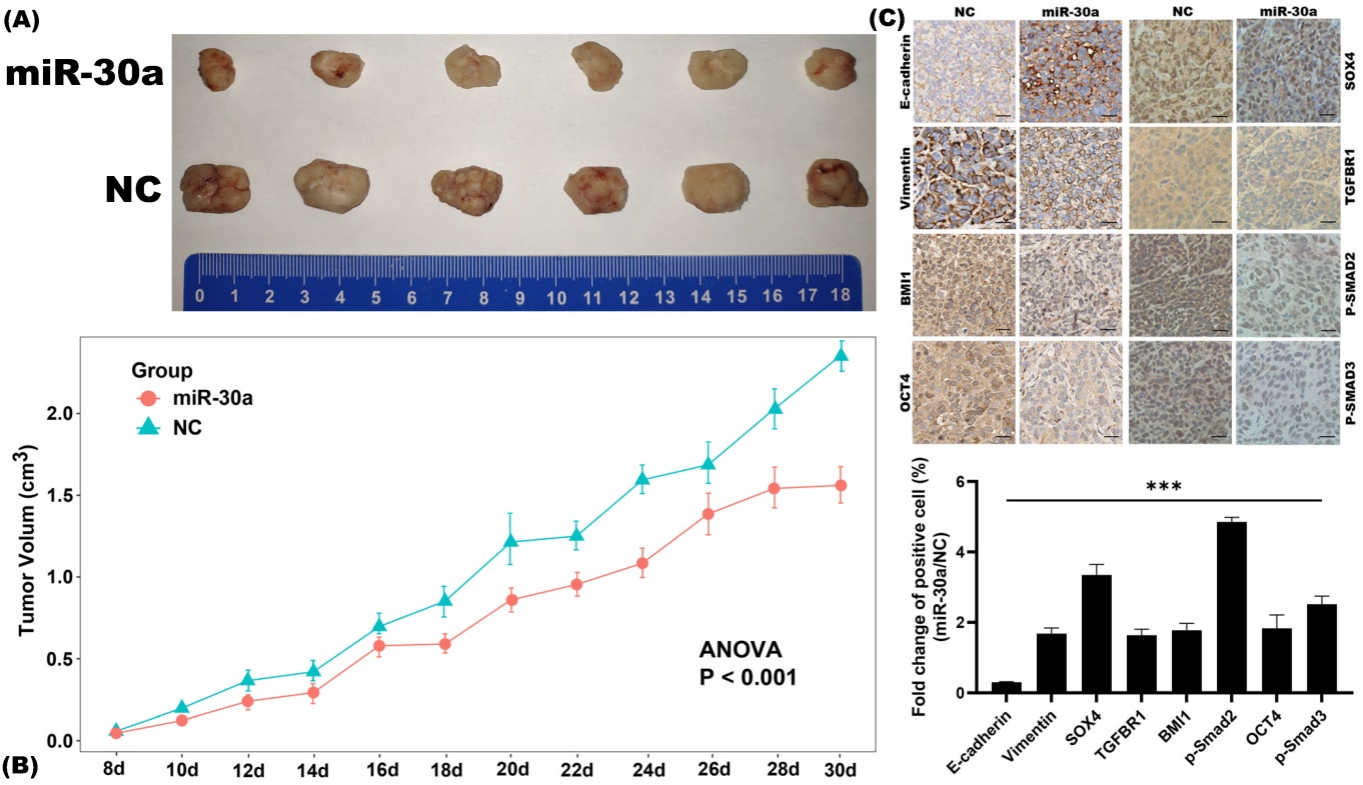
miR-30a inhibited EMT and CSC phenotypes in breast cancer *in vivo*. **(A)** Representative photographs of the xenograft tumors derived from subcutaneous implantation of MCF-7 miR-30a overexpressed and control cells on nude mice. **(B)** Growth curves of tumor. Values represent the mean ± SE. **(C)** Immunohistochemistry staining results of EMT-related, CSC-related, SOX4, and TGF-β/SMAD pathway related markers (× 200 magnification).

Subcutaneous xenograft nude mouse model was utilized to investigate the inhibitory effects of miR-30a on EMT and CSC phenotypes *in vivo*. We found that miR-30a significantly inhibited tumor growth as compared to the control group (Figure 6A-B). Further IHC staining revealed that miR-30a reversed EMT related proteins expressions and CSC markers (BMI1 and OCT4) and suppressed the expressions of SOX4 and TGF-β/SMAD pathway (phosphorylated SMAD2/3 protein and TGFBR1) *in vivo* (Figure 6C), as it did *in vitro.*

### Clinical significance of miR-30a and SOX4 in breast cancer

**Figure 7:**
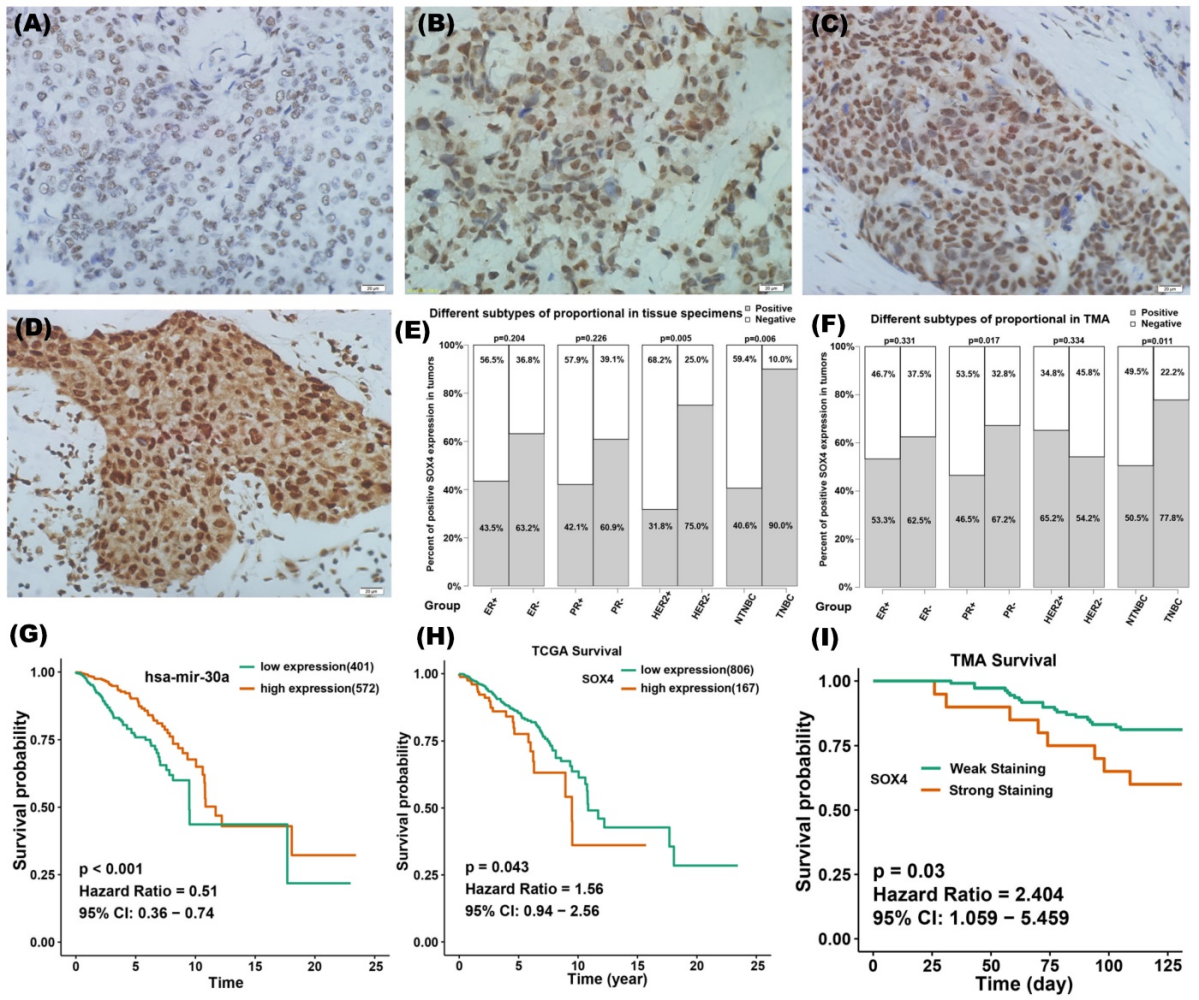
Clinical significance of miR-30a and SOX4 in breast cancer. **(A-D)** Representative images of immunohistochemistry staining of SOX4 on breast cancer samples at different immunoreactivity score (IRS): negative, weak, moderate and strong. **(E)** The relationship between SOX4 positivity and clinical characteristics in the group of 42 tumor specimens.** (F)** The relationship between SOX4 positivity and clinical characteristics in a tissue microarray containing 133 clinical samples. The red column represents positive proportion while the yellow one means negative proportion. **(G)** Survival curves of different expression groups of miR-30a in TCGA breast cancer database and the cutoff value was 16.87 for miR-30a expression. **(H)** Survival curves of different expression groups of SOX4 in TCGA breast cancer database and the cutoff value was 14.2 for SOX4 expression. **(I)** Survival curves of different expression groups of SOX4 from breast cancer tissue microarray.

IHC staining of SOX4 was performed on 42 tumor specimens and a TMA containing 133 samples. According to the IRS, the expression of SOX4 was assessed as being on one of four scales: negative, weak, moderate or strong (Figure 7A-D). Tumors with IRS of strong and moderate were defined as SOX4-positive and those with IRS of weak and negative were defined as SOX4-negativen. There were 52% (22 of 42) and 56% (75 of 133) positive staining of SOX4 in our clinical specimens and TMA, respectively. The relationship between SOX4 and clinical characteristics was analyzed (Figure 7E-F). Results showed that SOX4 expression was significantly correlated with PR status (*P* = 0.017 in TMA) and HER2 status (*P* = 0.005 in clinical specimens). The rate of SOX4-positive was significantly higher in TNBC than those in nTNBC samples (*P* = 0.006 in clinical specimens and *P* = 0.011 in TMA).

To evaluate the clinical significance of miR-30a and SOX4, TCGA database and IHC staining on tissue specimens were used. Bioinformatic analysis revealed that miR-30 high expression might be associated with a better survival (Figure 7G), whereas SOX4 high expression might be associated with a poorer survival (Figure 7H). Data from TMA verified that SOX4 expression was associated with poor prognosis in breast cancer (Figure 7I).

## Discussion

**Figure 8:**
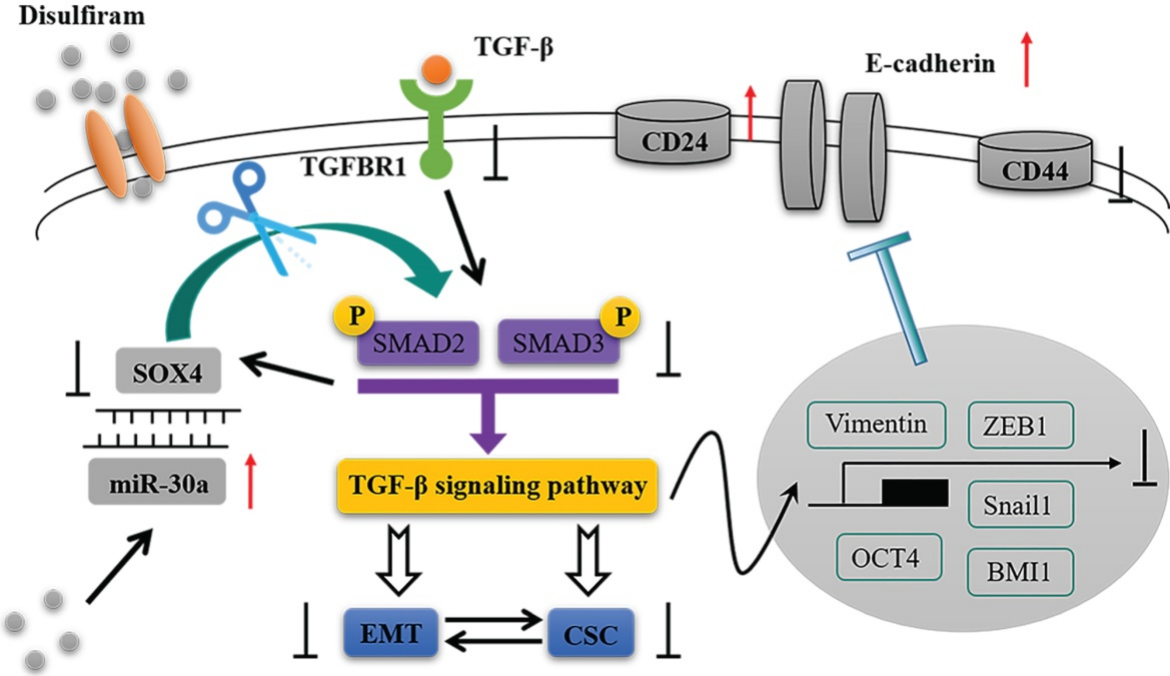
The schematic model of this study.

In the present study, we identified that miR-30a directly targets SOX4 and constitutes a double negative feedback loop (Figure 8). Overexpression of miR-30a or knockdown of SOX4 reversed EMT markers and inhibited stem cell-like phenotypes, as well as suppressing TGF-β/SMAD signaling pathway both* in vitro* and *in vivo*. The effects of miR-30a mediated were abolished by the reintroduction of SOX4. Furthermore, disulfiram, an old anti-alcoholism drug, is identified to modulate the miR-30a/SOX4 loop, by upregulating miR-30a expression. Based on TCGA database and clinical specimens, we found that miR-30a expression is associated with a good prognosis whereas SOX4 is associated with a poor prognosis for breast cancer patients. Our results suggest that the miR-30a and SOX4 double negative feedback loop is modulated by disulfiram and plays a key role in the regulation of EMT and stem cell-like phenotypes via TGF-β/SMAD signaling pathway in breast cancer.

Accumulating evidence links EMT program to the acquisition of stem cell-like (CSC) phenotype in cancer cells 5, 7, 29-31. Both EMT and CSC phenotypes may be involved in tumor development, therapy resistance, tumor progression, and tumor metastasis. MicroRNAs have served as dominant regulators of gene expression during tumorigenesis. More recent studies suggest miRNAs are involved in the regulation of EMT and CSC properties 32, 33. For example, members of the miR-30 family have been reported to inhibit EMT and CSC in different cancers. It has been revealed that, in breast cancer, miR-30a suppresses EMT progress by targeting ZEB2 34, Slug 35, Snail 36, ITGB3 37, and ROR1 38, and inhibits CSC properties by targeting Nanog 39, and AVEN 40. However, the exact mechanism of miR-30a in regulating the biological effects remains unclear.

Central to understanding miRNA function is the identification of miRNA targets 41. Since each miRNA has hundreds or thousands of potential targets, the accuracy of identifying functionally relevant target genes remains challenging 42. In our study, several independent approaches were adopted to identify novel miR-30a target genes including taking intersection of five prediction databases and GO analysis of candidate genes. Among all the predicted genes in Figure 2A, SOCS1 43, MYBL2 44, ITGB3 45, FOXD1 46 have been reported to be involved in the progression, metastasis or drug resistance in breast cancer. Especially, Snail has been reported to repress the transcription of E‐cadherin and SOX4 could regulate EMT with the same mechanism of Snail 47, they might have interaction through the process of EMT in breast cancer. Thus, we chose SOX4 for further experiments, and luciferase experiment was used to verify that SOX4 is a direct target of miR-30a and its expression is repressed by miR-30a. Previously, three studies have reported that miR-30a mediates cellular proliferation, apoptosis, and migration by targeting SOX4 in lung cancer 48, melanoma 49, and chondrosarcoma 50. As we know, a miRNA can repress its target gene. In some cases, the target gene can in turn regulate its regulatory miRNA. Several types of circuits exist involving regulatory of miRNA and its target genes. Ding et al. reported the miR-203 and SNAI2 double negative feedback loop plays important role in EMT 51. Bu et al identified the miR-34a and Numb forward loop to regulate stem cell division in colon cancer 52. Sylvestre et al. reported the miR-20a and E2F negative feedback loop may play a role in the regulation of cellular proliferation and apoptosis 53. Then, whether or how does SOX4 in turn regulate miR-30a? In this study, we firstly identified a double negative feedback loop between miR-30a and SOX4 when the induction of SOX4 suppressed the expression of miR-30a.

SOX4 is an important member of SOX family of transcription factors and participates in cancer development and progression by the transcriptional activation of downstream target genes in cancer-associated signaling pathways 54, such as PI3K pathway, Wnt pathway, and TGF-β pathway. SOX4 serves as a central component in TGF-β signaling pathway, which is induced by TGF-β and regulates TGF-β signaling by directly binding to SMAD3. SOX4 regulates TGF-β induced EMT by up-regulating the EMT-TFs, and maintains the property of CSC by inducing the expression of SOX2 55. In this study, the knockdown of SOX4 reversed EMT program and suppressed stemness properties as miR-30a did. The effects of miR-30a mediated were abolished by the restoration of SOX4. Although it has been reported that both miR-30a and SOX4 are involved in regulation of EMT and stemness, we are the first to report that miR-30a targets SOX4 to inhibit EMT and CSC phenotypes in breast cancer through TGF-β/SMAD pathway.

Disulfiram is an anti-alcohol drug that has been safely used for more than 60 years. Our previous study revealed that disulfiram can inhibit EMT and CSC phenotypes by suppressing ERK/NFκB/Snail pathway. Many other studies have also reported disulfiram serves as a novel anti-cancer drug by the regulation of cell growth, apoptosis, angiogenesis, and stemness 56, 57. In the present study, the expression of miR-30a, associated with a good prognosis of breast cancer, can be upregulated by disulfiram, which provide a new evidence for employing this old drug toward a novel anti-cancer use.

In summary, the miR-30a/SOX4 double negative feedback loop firstly identified here is modulated by disulfiram and regulates EMT and stem cell-like phenotypes through TGF-β/SMAD signaling pathway in breast cancer. These findings shed new insights into the molecular mechanism of EMT and CSC regulations, and thereby provide a novel therapeutic way for breast cancer treatment.

## Supplementary Material

Supplementary figures and tables.

## Abbreviations

EMT: epithelial-to-mesenchymal transition
CSC: cancer stem cells
BRCA: breast cancer
TNBC: triple negative breast cancer
nTNBC: non-triple negative breast cancer
DMEM: dulbecco's minimum essential medium
RPMI1640: roswell park memorial insitute 1640
miRNA: microRNA
miR-30a: microRNA-30a
mRNA: messenger RNA
EMT-TF: EMT-related transcription factors
qRT-PCR: quantitative real-time polymerase chain reaction
NC: negative control
shRNA: short hairpin RNA
UTR: untranslated region
TCGA: the cancer genome atlas
GSVA: gene set variation analysis
GO: gene ontology
TMA: tissue microarray
IHC: immunohistochemistry
TGF-β: transforming growth factor-β
SOX4: SRY-related HMG box 4
SMAD: drosophila mothers against decapentaplegic protein
ZEB1: zinc finger E-box binding homeobox 1
TGFBR1: transforming growth factor-β receptor 1
GAPDH: glyceraldehyde-3-phosphate dehydrogenase
BMI1: B cell-specific moloney murine leukemia virus integration site 1
OCT4: octamer-binding transcription factor 4
CD: cluster of differentiation
ALDH: aldehyde dehydrogenases
SE: standard error
IRS: immunoreactivity score
