# Supplementary material for: miR-30a/SOX4 Double Negative Feedback Loop is modulated by Disulfiram and regulates EMT and Stem Cell-like properties in Breast Cancer
Source: J Cancer. 2021 Jun 16;12(16):5053–65. doi: 10.7150/jca.57752 (PMC8247377; doi:10.7150/jca.57752)
Supplement: Supplementary file 1 — Supplementary figures and tables. [file jcav12p5053s1.pdf]

Supplementary materials

Figure S1 (A) Screening of miR-30a expression in breast cancer cell lines and normal breast cell line by qRT-PCR. *** P < 0.001. **(B)** qRT-PCR results of miR-30a in breast cancer cells stably transfected with miR-30a. *** P < 0.001. **(C) Relative expression of miR-30a in HBL-100 stably transfected with miR-30a inhibitor.** **(D)** Western blot results of expression for EMT related markers in HBL-100 cells transfected with miR-30a sponge. **(E)** The expression of miR-30a was downregulated in breast cancer in TCGA database. * P < 0.05.

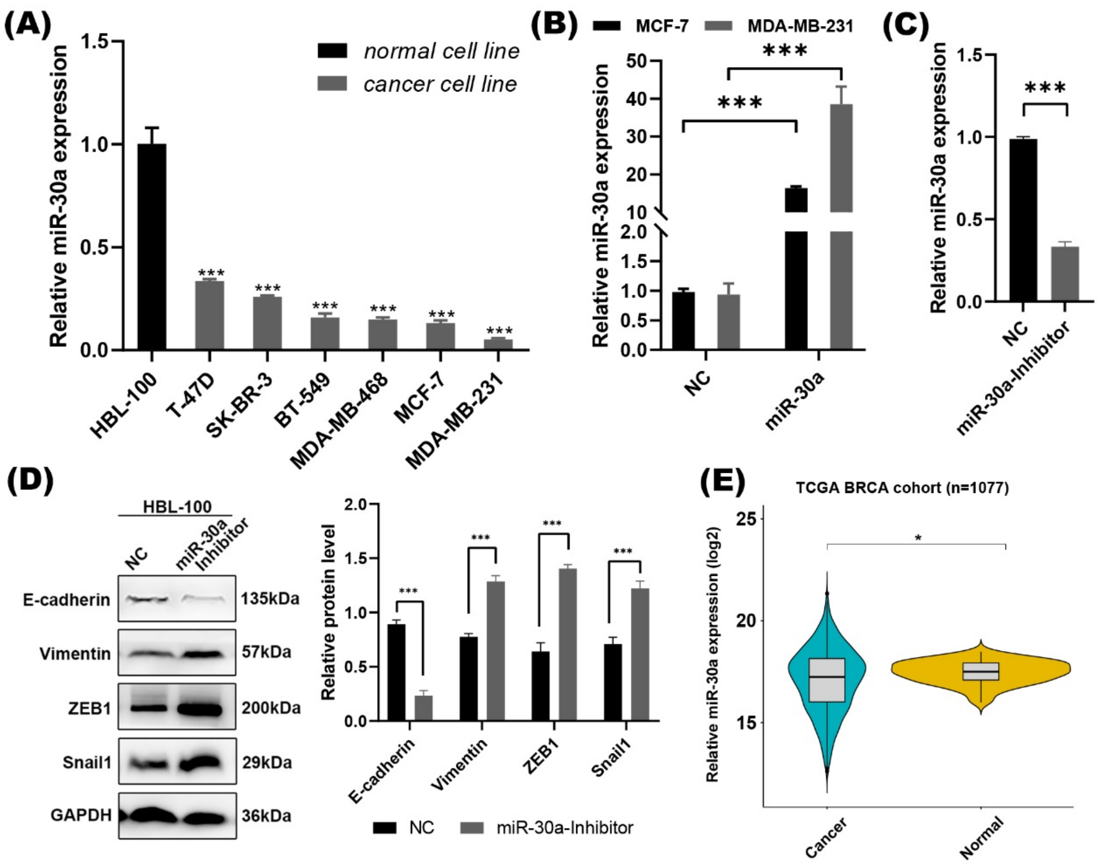

Figure S2 Screening of SOX4 expression in breast cancer cell lines and normal breast cell line. *** P < 0.001.

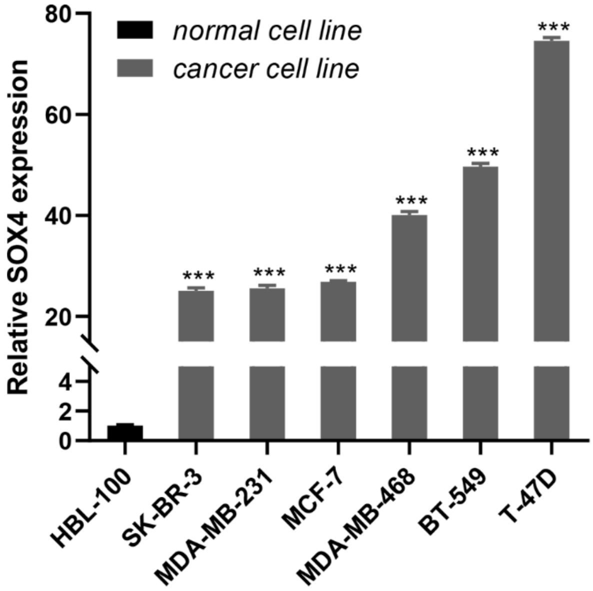

Figure S3 (A) qRT-PCR results of SOX4 expression in cells transfected with miR-30a or co- transfected with miR-30a and SOX4 lacking 3'UTR. **(B)** Western blot results of SOX4 expression in BT549 cells transfected with shSOX4. **(C)** Relative expression of SOX4 in BT549 stably transfected with shSOX4. **(D)** Western blot results of SOX4 and TGF-β related markers in HBL-100 cells transfected with miR- 30a sponge.

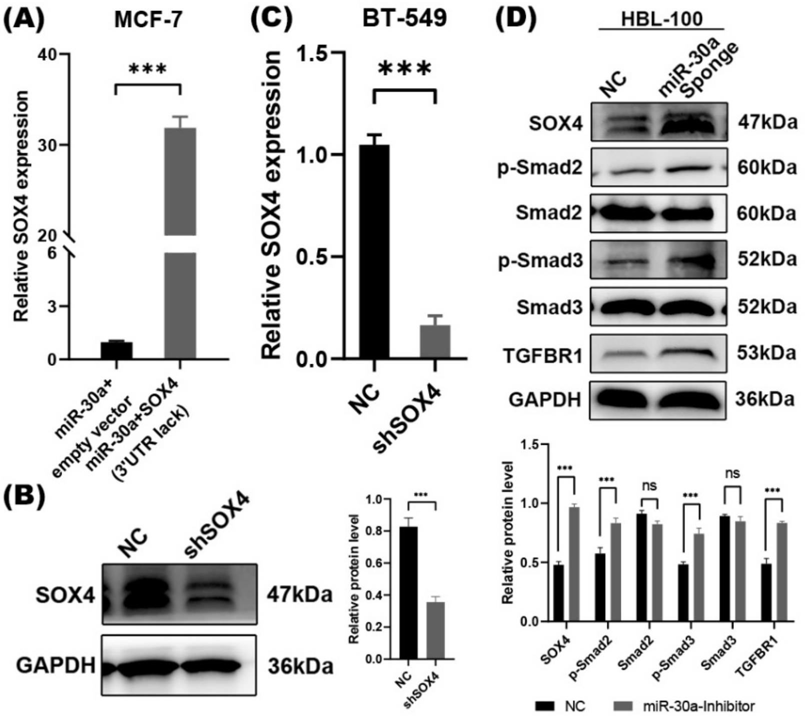

Supplementary table 1. Details for primary antibodies.

Antibodies	Working concentration			Sources
	WB	IF	IHC	
**E-cadherin**	1:1000	1:100	1:400	CST
**Vimentin**	1:1000	1:200	1:100	CST
**Snail1**	1:1000	-	-	CST
**ZEB1**	1:1000	-	-	CST
**SMAD2**	1:1000	-	-	CST
**p-SMAD2**	1:1000	-	1:200	CST
**SMAD3**	1:1000	-	-	CST
**p-SMAD3**	1:1000	-	1:200	CST
**TGFBR1**	1:500	-	1:100	R&D
**SOX4**	1:500	-	1:200	Abcam
**BMI1**	-	-	1:200	Proteintech
**OCT4**	-	-	1:200	Proteintech
**GAPDH**	1:3000	-	-	Servicebio

Abbreviations: WB: Western blot; IF: Immunofluorescence; IHC: immunohistochemistry; CST: Cell Signaling Technology (Beverly, MA, USA); R&D: R&D Systems GmbH, Germany.

Supplementary table 2. The information for specific primer sequences of genes.

Genes	Type	Primer sequences
**miR-30a-5p**	Forward	5’-CAGTGCAGGGTCCGAGGT-3’
	Reverse	5’-AACACGCTGTAAACATCCTCGAC-3’
**U6**	Forward	5’-CTCGCTTCGGCAGCACA-3’
	Reverse	5’-AACGCTTCACGAATTTGCGT-3’
**SOX4**	Forward	5’-CTGCGCCTCAAGCACATG-3’
	Reverse	5’-TTCTTCCTGGGCCGGTACT-3’
**GAPDH**	Forward	5’-GGGAGCCAAAAGGGTCATCA-3’
	Reverse	5’-TGATGGCATGGACTGTGGTC-3’

Supplementary table 3. Clinical characteristics of breast cancer patients.

	**Tissue specimens (n=42)**	**TMA (n=133)**
**Age, years (range)**	59 (26, 74)	55 (29, 87)
**ER Status**		
positive	23 (54.8%)	90 (69.2%)
negative	19 (45.2%)	40 (30.8%)
**PR Status**		
Positive	19 (45.2%)	71 (53.8%)
negative	23 (54.8%)	61 (46.2%)
**HER2 Status**		
Positive	22 (52.4%)	23 (17.7%)
negative	20 (47.6%)	107 (82.3%)
**Subtype**		
TNBC	10 (23.8%)	27 (20.8%)
Non-TNBC	32 (76.2%)	103 (79.2%)
**Tumor Size**		
⩽2cm	14 (32.6%)	52 (39.1%)
>2cm	29 (67.4%)	81 (60.9%)
**lymph node involved**		
0	22 (51.2%)	71 (53.4%)
1-3	16 (37.2%)	19 (14.3%)
>3	5 (11.6%)	43 (32.3%)

Abbreviations: TMA: tissue microarray; ER: estrogen receptor; PR: progesterone receptor; HER2: human epidermal growth factor receptor-2; TNBC: triple-negative breast cancer.
